# A Kirigami Approach of Patterning Membrane Actuators

**DOI:** 10.3390/polym13010125

**Published:** 2020-12-30

**Authors:** Harti Kiveste, Rudolf Kiefer, Rain Eric Haamer, Gholamreza Anbarjafari, Tarmo Tamm

**Affiliations:** 1 Intelligent Materials and Systems Lab, Institute of Technology, University of Tartu, Nooruse 1, 50411 Tartu, Estonia; deorell@gmail.com (H.K.); tarmo.tamm@ut.ee (T.T.); 2 Conducting Polymers in Composites and Applications Research Group, Faculty of Applied Sciences, Ton Duc Thang University, Ho Chi Minh City 700000, Vietnam; 3 iCV Research Lab, Institute of Technology, University of Tartu, 51011 Tartu, Estonia; eric@icv.tuit.ut.ee (R.E.H.); shb@ut.ee (G.A.); 4 Institute of Higher Education, Yildiz Technical University, Istanbul 34349, Turkey

**Keywords:** membrane actuators, PPy/DBS trilayer, bioinspired, simulation, patterning

## Abstract

Ionic electroactive polymer actuators are typically implemented as bending trilayer laminates. While showing high displacements, such designs are not straightforward to implement for useful applications. To enable practical uses in actuators with ionic electroactive polymers, membrane-type film designs can be considered. The significantly lower displacement of the membrane actuators due to the lack of freedom of motion has been the main limiting factor for their application, resulting in just a few works considering such devices. However, bioinspired patterning designs have been shown to significantly increase the freedom of motion of such membranes. In this work, we apply computer simulations to design cutting patterns for increasing the performance of membrane actuators based on polypyrrole doped with dodecylbenzenesulfonate (PPy/DBS) in trilayer arrangements with a polyvinylidene fluoride membrane as the separator. A dedicated custom-designed device was built to consistently measure the response of the membrane actuators, demonstrating significant and pattern-specific enhancements of the response in terms of displacement, exchanged charge and force.

## 1. Introduction

There is an increasing demand for smart devices with high functionality operating at low voltages. A lot of research has been carried out on conducting polymers, either for their flexibility [1] or conductivity, aiming at modern high tech applications, including but not limited to: sensors [2], super capacitors [3] and optoelectronics [4]. The other direction for such materials relates to mechanical work, such as in actuators, with an envisaged goal of recreating the behavior of natural muscles with so-called “artificial muscles” [5,6]. There have been relatively few works adapting biomimetic principles on bending actuators, like the recently shown reduction of friction by implementing a hydrophobic coating on bilayer rear side doubling bending displacement [7]. Another completely different direction of the biomimetic approach to the improvement of actuator behavior is based upon nature-inspired Kirigami designs, like the one taking after Vorticella convallaria [8], in various forms on helical actuators based on dielectric elastomers [9], or in shape memory alloys [10] or conducting polymers [11,12,13].

The typical application scenario of conducting polymers requires an electrolyte medium, as the positive charge formed on the polymer chains upon charging and removed by discharging is accompanied by the ingress (and expulsion) of ions with solvent (osmotic pressure [14]) to neutralize the charge, causing a volume change in the material. Bending displacement is achieved by attaching the conducting polymer as a layer on a non-conductive flexible layer on one side, forming a bilayer [15], or on both sides, making a trilayer [16]. Various designs of bending actuators have been proposed for robotic devices [17,18,19], smart textiles [20] and biomedical applications [21]. The most commonly applied conducting polymer is polypyrrole (PPy), often doped with dodecylbenzenesulfonate (PPy/DBS), which belongs among the cation-driven actuators, as the DBS^−^ anions are large enough to remain trapped in the PPy network [19]. The immobile DBS^−^ ions compensate for the positive charge of PPy^n+^ after synthesis and upon charging/oxidation (Equation (1), left side), while during reduction to PPy^0^, solvated (S) cations (C^+^) enter to in turn compensate for the charge of the anions, leading to a volume increase (swelling) upon reduction (Equation (1), right side) [22].

(1) $$\left[\left(PPy^{n+}\right)\left(DBS^{-}{)}_{n}\right)]+\;n\left(C^{+}\right)+m\left(S\right)+\;n\left(e^{-}\right)\;\begin{array}{c} \overset{\mathrm{red}}{\to }\\ \underset{ox}{\leftarrow } \end{array}[{\left(PPy\right)}^{0}{\left(DBS^{-}\right)}_{n}{\left(C^{+}\right)}_{n}(S{)}_{m}\right]$$

While amplifying the motion, bending laminate actuators are not the simplest to implement in applications; therefore, other alternative designs have been considered [23]. Membrane actuators, where the active film or laminate is supported perimetrically with a rigid frame and the mode of actuation is buckling, could be more practical in applications like micro pumps [24], but very few studies have been carried out on them—likely due to the small displacements achievable. However, a bioinspired kirigami approach has been successfully applied for increasing the displacement of ionic polymer composite sheet actuators [25].

Our goal in this work was to combine the well-established trilayer conducting polymer actuator material PPy/DBS with the kirigami approach of patterning the membrane with cuts of different computer-simulation-approved designs. The comparison of simulation and experimental results of conducting-polymer-patterned membranes is shown here for the first time.

The bending displacement of the membrane actuators was measured by a laser displacement meter in a custom-designed device to obtain reliable data. Square wave potential step measurements were performed and the PPy/DBS trilayer was characterized by scanning electron microscopy (SEM), energy dispersive X-ray spectroscopy (EDX) and conductivity measurements.

## 2. Materials and Methods

### 2.1. Materials

Polyvinylidene fluoride membranes (Durapore PVdF, 100 µm thickness, hydrophilic, pore size 0.1 µm) were purchased from Merck (Darmstadt, Germany) and used as supplied. Polyoxymethylene (POM, granule 3 mm), sodium dodecylbenzenesulfonate (NaDBS, 99%), ammonium persulfate (APS, 98%), ethylene glycol (EG, 99.8%), ethanol (EtOH, technical grade) and bis(trifluoromethane)sulfonimide lithium salt (LiTFSI, 99.95%) were obtained from Sigma-Aldrich and applied without further purification. Pyrrole (Py, 98%, Sigma-Aldrich, Taufkirchen, Germany) was distilled under reduced pressure and stored in the dark at −20 °C under nitrogen. Milli-Q+ (deionized water, Tallinn, Estonia) was applied as a solvent.

### 2.2. Electropolymerization and Pattern Design

**Scheme 1:**
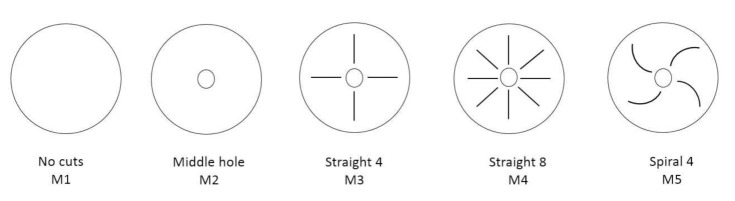
Membrane designs. No patterning: M1; patterned: M2 (middle hole, diameter 4 mm), M3 (a hole and four straight cuts with a 2 mm space between the hole and the cuttings), M4 (a hole and 8 straight cuts), M5 (a hole and spiral cuts).

The PVdF membranes (length 5 cm × width 5 cm, thickness 100 µm) were coated with PPy over chemical polymerization, as described previously [26]. PVdF membranes were coated on both sides with pyrrole monomer and dipped in aqueous oxidant solution (0.075 M APS, 0.005 M NaDBS) for 30 s. After coating, non-bound PPy particles were mechanically rubbed off by hand (using latex gloves) and the PPy-coated PVdF washed several times with ethanol to remove excess pyrrole and with Milli-Q+ to remove residual oxidant solution. The conductive PVdF membranes were then dried in an oven at 60 °C at 2 mbar for 12 h. Electropolymerization of pyrrole on the coated PVdF sheets as the working electrode between two stainless steel nets as the counter electrodes (two electrode cells) was performed galvanostatically (0.1 mA cm^−2^ at −5 °C for 4000 s) controlled by a postentiostat/galvanostat (PARSTAT 2273, Princeton applied research, Oak Ridge, TN, USA) in a monomer solution containing 0.2 M pyrrole and 0.2 M NaDBS. The obtained PPy-PVdF-PPy trilayers (M1) were cut with a scalpel in a ring shape (diameter 4 cm) using a template washed with ethanol and Milli-Q to remove excess monomer and NaDBS and dried (40 °C, 2 mbar, 12 h) in the oven. The patterning and labels of the samples are shown in Scheme 1.

The cutting was performed with a scalpel from outside to inside, making sure no short circuit or damage to the membrane actuators was made.

### 2.3. Fabrication and Actuation

**Scheme 2:**
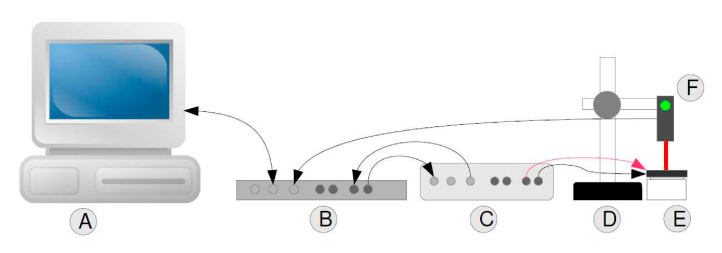
In-house data acquisition system. (**A**) computer with LabView, (**B**) data acquisition system (SC-2345), (**C**) current amplifier, (**D**) stand for the distance sensor, (**E**) measurement device and (**F**) distance sensor.

To obtain a solid construction in which the membrane-based actuator (PPy-trilayer) could be measured, a custom device was constructed. The membrane actuator was placed between solid stainless steel rings (height, 1 cm; width of ring, 1 cm; diameter, 4 cm) that functioned as the working- and counter-electrode contacts, respectively, but also kept the membrane in place. The body of the device was made by lathing (ZHMAC, Machine WMP290V-F, Nantong Zongheng Machinery Technology Co, Nantong, China) a cylindrical shape into a 4.1 cm diameter with a depth of 2.0 cm from a solid POM block. The measurements (Scheme 2) were performed using a Lab View program (Austin, TX, USA) (A) controlling an NI-2345 data acquisition system (B) (National Instrument corp., Austin, TX, USA) that provided the driving signals through a current amplifier (C) (ACM Instruments, Cumbria, UK) as well as simultaneously recorded the bending displacement in the measurement device (E) with a fixed (D) optical distance sensor (F) (Keyence, LK-G10, Itasca, IL, USA), with displacement automatically calculated from the distance change.

To get a reliable signal from the optical sensor, a black POM gasket (3D-printed, Tronxy P802E 3D Printer kit, Shenzhen Tronxy Technology Co., Shenzhen, China) was glued onto the membrane actuator. The gasket also functioned to consolidate the 2 mm ring between the pattern and hole. Square wave potential step measurements (±0.7, 1.67 mHz to 1 Hz) were performed in 1 M LiTFSI-aq electrolyte. From chronoamperometric measurements at each applied frequency, the diffusion coefficients upon oxidation (D_ox_) and reduction (D_red_) were calculated over Equations (2) and (3).

(2) $$ln\left[1-\frac{Q}{Q_{t}}\right]=-bt,$$

(3) $$D=\frac{bh^{2}}{2},$$

Q is the charge at each time point obtained from the integration of the current density curves, and Q_t_ is the total charge. Plotting the left term of Equation (2) against time, the slope b was obtained [27]. The diffusion coefficients were calculated following Equation (3), using thickness h (here the thickness of the deposited PPy/DBS plus that of the chemical PPy, 5 µm in total).

Before the measurements commenced, each membrane actuator was soaked for 24 hours in the electrolyte solution. Three different samples were independently polymerized and measured; the results are given as mean values with standard deviations.

### 2.4. Characterizations

The M1 samples were characterized using scanning electron microscopy (SEM, Helios NanoLab 600, FEI, Hillsboro, OR, USA) and the ion content on the surface was determined over EDX spectroscopy (Oxford Instruments with X-Max 50 mm^2^ detector, Concord, MA, USA) in charged (0.7 V, 5 min) and discharged (−0.7 V, 5 min) state. The conductivity of the membrane actuator was determined using a four-point-probe conductivity meter (Model RM2, Jandel 4-Point Probe Head, Leighton Buzzard, UK).

## 3. Results and Discussion

### 3.1. Device Design

**Figure 1:**
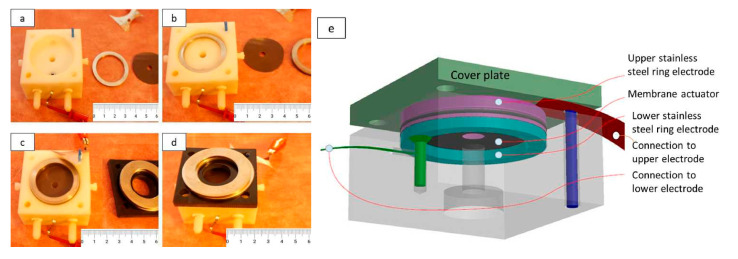
Assembly steps of the measurement device (scale bar 6 cm) (**a**–**d**) with an M2 membrane: the lower electrode as the counter-electrode and the upper electrode as the working electrode. A schematic view of the device with the electrodes, the membrane actuator and the connections in place (**e**).

Reliable measurements of membrane actuators require a specific holder to fulfill certain requirements of easy handling, clamping the membrane actuator without a short circuit and avoiding leakage of electrolytes. In order to avoid complicated arrangements, a simple approach with stainless steel rings on either side of the membrane gave the best results. Figure 1a–d show the step-by-step assembly of the device with an M2 actuator, with Figure 1e as the schematic of the device.

The main components can be seen in Figure 1a, starting from the POM embodiment on the left, followed by the lower steel ring, which is already in place in Figure 1b. Here, the membrane actuator is waiting for its turn; it has been placed onto the lower steel ring in Figure 1c and is already held in place by the upper steel ring. In Figure 1d, the cover plate is also in position, weighed down by two heavy steel rings.

### 3.2. Characterization of Membrane Actuators

**Figure 2:**
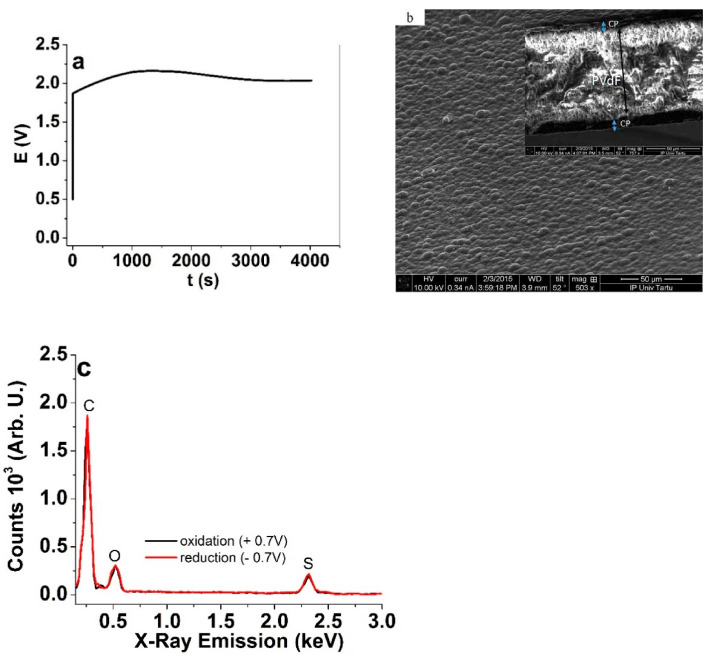
(**a**) The galvanostatic polymerization curve (two-electrode cell) of polypyrrole (PPy) on a chemically PPy-coated polyvinylidene fluoride (PVdF) membrane at −5 °C, (0.1 mA cm^−2^, 4000 s); (**b**) SEM micrographs (scale bar 50 μm) of the surface of an M1 actuator with the cross section in the inset showing arrows of PVdF and the deposited conducting polymer. (**c**) EDX surface spectra of M1 after actuation, oxidized at +0.7 V (black curve) and reduced at −0.7 V (red curve).

The chemically polymerized PPy in 1.5 μm thickness on either side of the PVdF membranes had a conductivity of 24 ± 2 mS cm^−1^. These PPy layers were applied as the working electrode(s) in the following galvanostatic electropolymerization (Figure 2a) of pyrrole. The SEM images of PPy-M1 are presented in Figure 2b. The ion content in the surface of M1 was determined with EDX measurements (Figure 2c) after the actuation cycles in an oxidized state (5 min polarization at +0.7 V) and a reduced state (5 min, −0.7 V).

The potential time curve in Figure 2a of the galvanostatic polymerization revealed a voltage increase until 1600 s reaching 2.6 V, with a decrease thereafter, dropping to 2.03 V by the end the polymerization. The increase was likely caused by the increased surface roughness (area) while the decrease was related to the increased conductivity as the layer grew in thickness. The deposited PPy/DBS on the PPy(chem)-PVdF-PPy(chem) membrane (Figure 2b) revealed the typical cauliflower surface morphology of PPy [28]. The cross-section image shows the PVdF membrane in the middle with PPy/DBS layers in the range of 3.5 ± 0.3 µm on each side. The electronic conductivity of the samples directly after polymerization in the oxidized state at 0.7 V was 43.3 ± 4.1 S cm^−1^.

The surface EDX spectra (Figure 2c) revealed signals of carbon (C) at 0.27 keV, oxygen (O) at 0.52 keV and sulfur (S) at 2.32 keV. No significant changes can be observed between the oxidized and reduced states, which confirms that the (here undetected) Li^+^ cations were the mobile species during the redox cycles, as the DBS^−^ content (represented by the oxygen and sulfur peaks) did not change [29].

### 3.3. Displacement of Membrane Actuators

#### 3.3.1. Simulation of Bending Displacements

**Figure 3:**
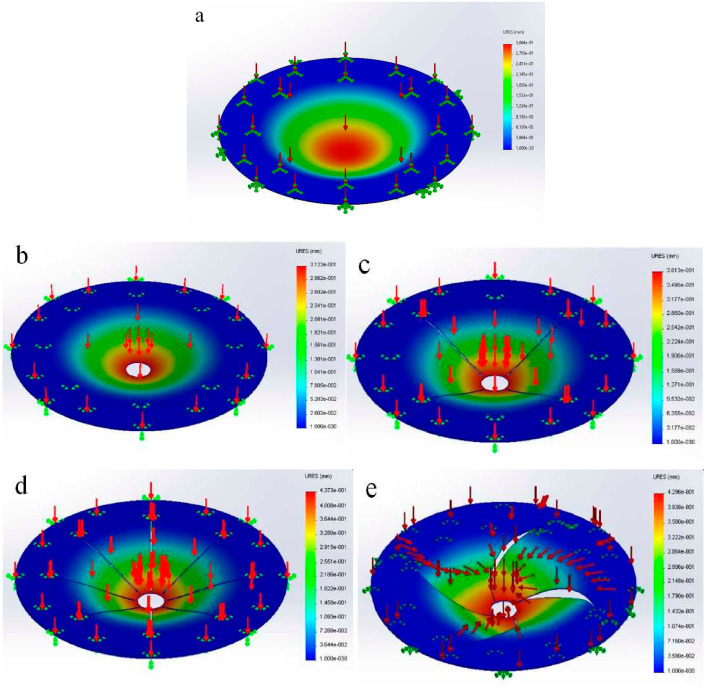
Finite element simulations of deflections (extant as color scales) resulting from a uniform constant pressure of 0.7 kPa on the membranes (**a**) M1, (**b**) M2, (**c**) M3, (**d**) M4 and (**e**) M5. The arrows on the membrane symbolize the vertical pressure on each point of the membrane. The green dotted points refer to the part of the membrane being fixed (1 cm from the edge), as under the experimental conditions.

The responses of the membranes to uniform vertical pressure (0.7 kPa) were simulated using finite element analysis as implemented in Solidworks [30], applying the large displacement solution. The Timoshenko beam model adapted to circular plates was applied, with the simplifications of isometric membranes and limiting to small trains to endure linearity between stress and strain [31]. Similarly to the experiments, the membranes in diameters of 4 cm were considered, with 1 cm fixed (the same as the stainless steel rings’ positioning in the experiments). Figure 3a–e show the simulated heatmaps of maximum bending displacements of membranes M1–M5.

The simulations considered the situation of two conduction polymer layers producing an equal force on the isometric membrane, working against the Young’s modulus of PVdF of 2.0 GPa (the modulus of CP itself was neglected). The effect of the electrolyte solution present in the experiment was also neglected in the theoretical model calculation in order to simplify the approach. The displacements, according to the simulations for M1–M5, were 276 μm, 312.2 μm, 361.3 μm, 437 μm (highest) and 429.6 μm, respectively. To test out the simulation results, membranes with the same patterns were studied experimentally; the results are analyzed in the next section.

#### 3.3.2. The Improvement of Displacement by Patterning

**Figure 4:**
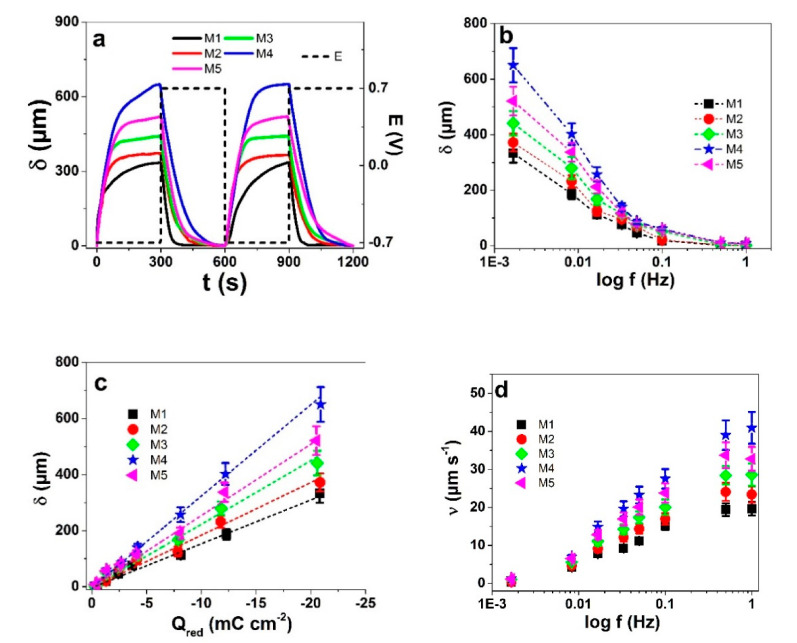
Square wave potential step (16.7 mHz, ± 0.7 V, dotted line) measurements in bis(trifluoromethane)sulfonimide lithium salt (LiTFSI-aq) electrolyte. (**a**) Two subsequent cycles (4th to 5th) of displacement δ response of membrane actuators M1 (black line) and patterned M2 (red), M3 (green), M4 (blue) and M5 (pink). (**b**) Displacements against log frequency of M1 (■), M2 (●), M3 (♦), M4 (★) and M5 (◄); and (**c**) displacements against charge density upon reduction. (**d**) The actuation speed ν of M1–M5-type membrane actuators against log frequency. The dashed line in (**c**) represents the linear fit and is shown here only for orientations.

For each type of membrane actuator, three different samples were fabricated. The results are presented as mean values. In general, the displacement of a membrane actuator is lower than that of a freely bending trilayer actuator due to their lack of freedom of motion, but patterning was expected to improve the displacement. Square wave potential steps were used as the driving signal to study the displacement response of membrane actuators M1–M5, with two subsequent cycles of displacement against time at 1.67 mHz, presented in Figure 4a. The displacements against the applied frequency (1.67 mHz to 1 Hz) are shown in Figure 4b and the displacements against charge density are presented in Figure 4c. The actuation speed of membranes is shown in Figure 4d.

The unmodified reference membrane actuator M1 showed displacement (Figure 4a) upon reduction in the range of 334 µm while M2 (perforated in the center) had a displacement of 371 µm, an increase of 10% over the M1 type. With further patterning, the displacements of the actuators M3 to M5 reached the highest for the M4 pattern. The main actuation mechanism relates to Equation (1), as the immobility of the DBS^−^ ions in PPy/DBS forces the solvated Li^+^ cations to enter the film during reduction to achieve charge neutrality, leading to volume expansion [32].

M4 showed the highest displacements independent of the frequency, which indicates that the electro-chemo-mechanical process is the same for all actuators, while just the freedom of motion differs. The same is further confirmed by the displacement–charge density plots (Figure 4c). As the order of charge densities was the exact same as that of displacements, it can be concluded that the confined/suppressed motion of the less-patterned membranes also suppresses the charge transfer, as the ions compensating for the charge cannot be transported efficiently. With increasing charge density, the membrane displacements increased linearly, confirming the Faradaic actuation process [5,33].

For potential application of membrane actuators, the actuation speed is also an important parameter (Figure 4d). The highest rate was again shown by M4 driven at 1 Hz with nearly 40 µm s^−1^. The other patterns were less efficient in reducing the material geometry backpressure with rates peaking off at lower driving frequencies. Hence, larger displacements and higher speeds are closely related in the case of membrane actuators.

The comparison of the measured actuation results at two frequencies (1.67 mHz and 16.7 mHz) with the simulation (constant 0.7 kPa stress), both relative to the M1 non-patterned membranes, are shown in Table 1.

Overall, the simulations qualitatively predicted the correct order of the pattern performance. The theoretical improvement over M1 from the simulations of M2 was found to be in the range of 11.6%, which agrees well with the 10–11% improvement observed in the experimental studies (Table 1). Interestingly, for the M3 and M5 patterns, the predicted relative improvements over M1 agree very well with the experimental observations at 1.67 mHz, while the improvements were clearly higher at 16.7 mHz. Even more surprisingly, the improvements measured for M4 were significantly higher than the predicted ones at both frequencies. Moreover, while there was little between the predicted performance of M4 and M5 in the simulations, M4 dominated in the experiments.

**Figure 5:**
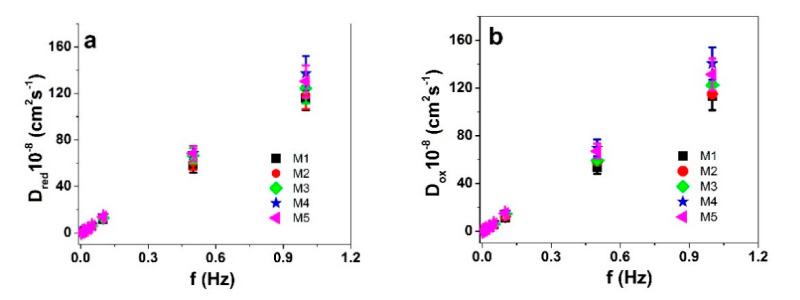
Diffusion coefficients obtained from Equations (2) and (3), from square wave potential step measurements in LiTFSI-aq electrolyte in a potential range of ± 0.7 V. From the membrane actuators M1 (■), M2 (●), M3 (♦), M4 (★) and M5 (◄), the diffusion coefficients upon reduction D_red_ are shown in (**a**) and those upon oxidation D_ox_ are presented in (**b**) against the applied frequency f.

The unpredicted strong performance improvement by the best patterns at the higher frequency can be explained by several factors, one of which is the missing electrolyte solution medium (in the experiment, LiTFSI-aq). The membrane actuators were imposed with solvent uptakes over osmotic pressure [14] and solvated Li^+^ ions enter the PPy/DBS membrane on reduction (Equation (1)). At higher frequencies, the penetration of the charge and the counterions into the material is smaller than that at lower frequencies; hence, even minor improvements to the freedom of motion of the matrix generate relatively larger effects on the response. Moreover, the ingress of solvated ions can generate somewhat of a snowball effect, where the polymer swells and expands, and creates space for the consecutive ions to enter. The effect of the increased deformability of the material to ion mobility inside can be quantified in terms of diffusion coefficients, obtained from Equations (2) and (3). The results are presented in Figure 5a,b.

As seen before [34], the diffusion coefficients for both oxidation and reduction processes increased nearly linearly with increasing frequency (Figure 5). The electroactive PPy/DBS underwent relaxation processes upon reduction, for which at higher frequencies a shorter time is available, meaning a “shallower” ion penetration, lower amount of exchanged ions and lower charge density. At low frequencies, all diffusion coefficients upon reduction and oxidation appeared to be very similar, while they became more and more different with increasing frequency. The order of the diffusion coefficients was the same as those of the displacements or the charge densities, showing that increased ion mobility inside the films resulted from the patterns as the overall deformability of the material was improved. The effect is more pronounced at higher actuation rates, as only the most easily accessible locations can participate at higher frequencies—the ones most affected by the patterning.

**Figure 6:**
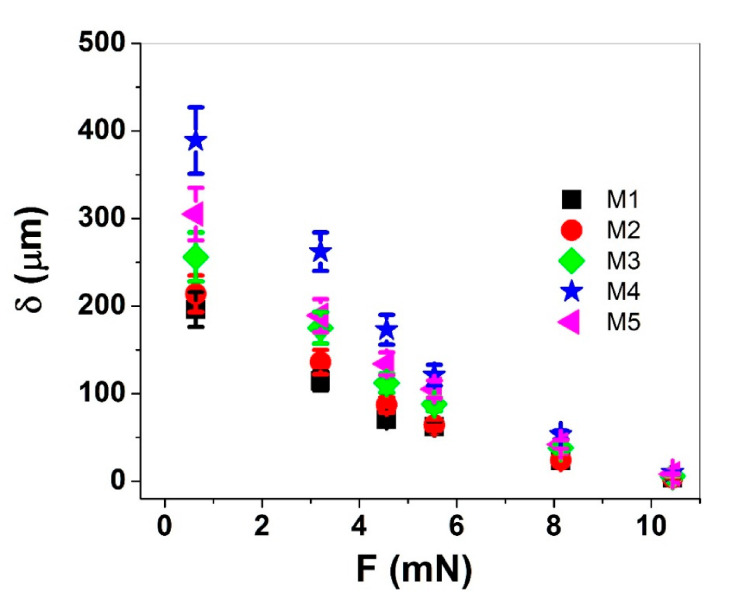
Displacements of membrane actuators M1 (■), M2 (●), M3 (♦), M4 (★) and M5 (◄) with different loads (counter-forces) driven at 8.33 mHz at ± 0.7 V in aqueous LiTFSI electrolyte.

An actuator is most useful when it can actually do some useful work, e.g., lift a weight. To investigate the performance during lifting, a special cylindrical tube was 3D-printed from POM (inner diameter, 5 mm; length, 25 mm; and wall thickness, 2 mm) and placed on the membrane’s inner ring. The tube reached out of the electrolyte solution (limiting the effect of the buoyant force) and was glued around the central hole of the M2–M5 membrane actuators (just the center for M1). The weight of the hollow tube was 65 mg (0.64 mN). With the additional weights in place, the total loads were 351 mg (3.2 mN), 465 mg (4.56 mN), 565 mg (5.54 mN), 831 mg (8.14 mN) and 1065 mg (10.44 mN), respectively. Figure 6 demonstrates the comparative results of M1–M5 membrane displacements driven at 8.33 mHz with different loads applied.

With increasing counter-force, the displacement of the M1–M5 membrane actuators decreased, whereas the M4 membrane reached nearly double displacement compared with M1 and M2 with all of the different counterforces applied.

Therefore, the increased exchanged charges and displacements of the more substantial M4 patterning did not come at the cost of reduced force or work capacity, as the patterned membrane actuator was still capable of lifting the same weights higher than the non-patterned M1 and M2. Therefore, the force and work capacity not required to overcome the rigidity of the membrane itself could be spent on lifting payloads. In summary, virtually every aspect of the membrane actuator’s performance was increased with cuts of the best patterns. Thus, the approach is envisaged for applications in pumps, valves, liquid lenses and robotic motion. Further research as well as optimization will be performed in the future to tune the properties of the actuators to particular applications. Additionally, future adaptation of bending membranes with geometrical diversity affecting the overall performance can be made. As for the simulations, other models like those of pressurized circular elastic membranes [35] could be adapted over the bending beam models with a patterned structure to improve the prediction accuracy for the patterns.

## 4. Conclusions

The performance of ionic electroactive polymer membrane actuators is strongly limited mechanically by the lack of freedom of motion. Already very simple patterns in the form of cuts in the membrane can increase the freedom of motion (and hence, the displacement) significantly. Computer simulations suggested that other more elaborate designs would bring about an additional performance increase, and the results were verified using membrane actuators based on PPy/DBS-PVdF-PPy/DBS trilayers. A special design of the device that held the membrane actuator in place was introduced that allowed for measurements of the displacements by a laser displacement meter. The experiments supported the modeling results, as all of the patterns clearly increased the performance. The highest displacements were achieved by the most-patterned M4 membrane (a center hole and eight straight cuts) in a range of 49–56% over the M1 non-patterned membrane, depending on the driving frequency. The analysis of charge densities and diffusion coefficients demonstrated that the rigidity of the membrane suppressed both charge transfer and the related ion flux; both were significantly improved by the patterning. As the M4 membrane actuator also had improved the actuation rate, and was the most capable in lifting different weights, showing the best work capacity, there appear to be no drawbacks to the patterning approach. For the envisaged applications in membrane pumps, liquid lenses, soft robotics, etc., dedicated optimizations in membrane size, rate of actuation and other patterns can further improve the performance.

## Figures and Tables

**Table 1:** Simulated and experimental displacements (% improvement over the M1 membrane) of membranes with different cutting designs (M1–M5, Scheme 1) (relative to M1).

Membrane Actuator Pattern	Relative Improvement, Simulation	Relative Improvement, Experimental (1.67 mHz)	Relative Improvement, Experimental (16.7 mHz)
M1	0	0	0
M2	+11.6%	+10%	+11%
M3	+23.7%	+24.4%	+33.1%
M4	+37%	+48.7%	+56.2%
M5	+36%	+36%	+41.1%

## Data Availability

The data presented in this study are available on request from the corresponding author. The data are not publicly available due to ongoing studies.
